# Reliable Data Collection Methodology for Face Recognition in Preschool Children

**DOI:** 10.3390/s22155842

**Published:** 2022-08-04

**Authors:** Hye-min Won, Hyeogjin Lee, Gyuwon Song, Yeonghun Kim, Nojun Kwak

**Affiliations:** 1 Department of Electrical and Computer Engineering, Ajou University, Suwon-si 16499, Korea; 2 Graduate School of Convergence Science and Technology, RICS, Seoul National University, Seoul 08826, Korea; 3 Advanced Institute of Convergence Technology, Suwon-si 16229, Korea; 4 Robotics Planning Team, Hyundai Motor Company, Uiwang-si 16082, Korea

**Keywords:** face dataset, face database, children’s face data, children’s face database, optimal camera setup

## Abstract

Most face datasets target adults who can make their own decisions. In the case of children, consent from parents or guardians is necessary to collect biometric information, thus making it very difficult. As a result, the amount of data on children is quite small and inevitably private. In this work, we built a database by collecting face data of 74 children aged 2–7 years in daycare facilities. In addition, we conducted an experiment to determine the best location to perform face recognition on children by installing cameras in various locations. This study presents the points and methods to be considered to build a children’s face dataset and also studies the optimal camera installation setups for the face recognition of children.

## 1. Introduction

As biometrics are actively applied to various parts of real life due to the development of deep learning, numerous biometric data are collected and applied. Among these, children’s face image data is an important one that can be used in preventing crimes against children and providing various welfare benefits. However, in the case of children’s data, the amount is very small and its use is limited.

Most face datasets target adults who can make their own decisions. In the case of an adult, they can decide whether to provide biometric data by themselves by recognizing the precautions for providing biometric data. However, in the case of children, it is difficult for them to recognize the precautions for providing biometric data on their own, so the consent of parents or guardians is absolutely necessary to collect data. Legal protection of children’s information prioritizes parental control and consent [1]. A child’s right to privacy should not be subject to the wishes and actions of others or the control and consent of others. Therefore, even after parental consent, all information should be kept confidential to protect children’s privacy in principle.

With the changing parenting environment, due to the low birth rate and the increase in double-income families, the role of childcare facilities has increased. Therefore, this study focused on attendance check among methods to prevent child safety accidents. Attendance check is one way to prevent safety-related accidents in a child care center. When a daycare center provides transportation as well as education, teachers should check the attendance of children before and after boarding. At this time, automatic attendance checks can not only serve a safety and security role, but also provide a reduction in teachers’ work burden. In this study, we collected a database of children’s faces that can be used in a real-time automatic attendance system.

Srinivas et al. experimented with deep learning face recognition systems to see if there was a decrease in performance in the case of children when compared with the results obtained for adults [2]. The conclusion of that study was that the use of children’s data in an adult-targeted system resulted in performance degradation. Through this experiment, it can be seen that children’s face recognition requires a system dedicated to the children’s face dataset and the corresponding environment. In collecting children’s face data, various considerations must be taken into account. These include the children’s age, children’s concentration, and various environmental factors. In addition, as children’s faces change as they grow, continual data collection is required to match the growth rate of children. In this study, we present the points and methods to focus on while building a database for children, and examine effective camera installation location for face recognition of children.

Face recognition of children is an important research project that can be used in preventing crimes against children and providing various welfare benefits. For children’s face recognition, a database containing the children’s face image data is absolutely necessary. However, because of the world-wide strengthened Personal Information Protection Act, the preparations and procedures for collecting face data have become very complicated. In 2020, datasets containing personal information such as LFR [3], RMFRD [4,5], and SMFRD [4,5] were collected. Most of these datasets are targeted at adults or celebrities. In addition, most of the databases do not contain newly collected data; rather, they are composed by merging or modifying existing data such as LFW [6], CFP [7], and CASIA-WebFace [8].

In this study, we present the points and methods to focus on while building a database for children, and determining an effective camera installation location for face recognition of children. A database was created by collecting the face data of 74 children, aged 2 to 7, at a daycare facility, and an experiment was conducted to perform face recognition by installing cameras in various environments. We have improved the reliability of the data collected by our method through experiments.

We collected children’s data to the extent permitted by the Republic of Korea’s laws. All data were collected with the prior consent of the children’s parents, guardians, and teachers. The data collected is used only for this study to protect children’s privacy and is not publicly disclosed.

## 2. Related Work

There are many datasets for face recognition [9,10,11,12,13,14,15,16,17,18]. A face dataset can be divided into five categories, depending on whether it is public or distributed, and whether the photographed party agrees to photograph (Table 1).

**Table 1:** List of children’s faces datasets.

Category	Dataset	Age Group	Availability	Consent	The Number of Subjects	The Number of Images
1	MORPH [9]	16–77	Public	o	70,000	402,055
	MORPH (Academic) [9]	16–77	Public	o	13,618	55,134
	FG-NET [10]	0–69	Public	o	82	1002
2	PCSO [11,12]	18–83	Private	x	18,007	147,784
	MSP [11]	18–78	Private	x	9572	82,450
3	CLF [13]	2–8	Private	Parental agree	919	3682
	NITL [14]	0–4	Private	Parental agree	314	3144
	Newborns [15]	Newborn	Private	Parental agree	450	1200
4	CMBD [16]	2–4	Public	Parental agree	106	1060
5	CACD [17]	16–62	Public	A tacit agreement	2000	160,000
	ITWCC [18]	5–32 months	Public	A tacit agreement	304	7990
6	Ours	2–7	Private	Parental agree	78	Video 35:59

First, there are those that disclose and distribute the dataset with consent from those who provide face data. This includes the MORPH dataset, FG-NET dataset, and FaceTracer dataset. The MORPH Longitudinal Database consists of two core datasets, commercial and developmental. It contains approximately 400,000 face images of people between the ages of 16 and 77 [9]. The face and gesture recognition research network (FG-NET) dataset consists of a total of 1002 face images of 82 people between the ages of 0 and 69 [10]. It is probably the only public dataset that provides face data for infants and children, but it has only 400 face images of children under the age of 15.

Second, there are those datasets that have been created without the consent of the person providing face information due to social needs, but that do not disclose or distribute it. The Michigan State Police (MSP) dataset and Pinellas County Sheriff’s Office (PCSO) dataset are this type of dataset. The MSP dataset contains 82,450 face mugshots of 9572 habitual offenders from 2002 to 2015 [11]. The PCSO dataset contains 147,784 face mugshots of 18,007 habitual offenders from 1994 to 2010 [11,12].

Third, there are face datasets of minors, which include face data obtained with the consent of their guardians, but the data are not disclosed or distributed. This includes the CLF dataset, NILT dataset, and extended newborns face database. The Children Longitude Face (CLF) dataset consists of 3682 face images of 919 children [13]. The Newborns, Infants, and Toddlers (NITL) dataset consists of 2144 face images of 314 children [14]. The Newborns Face Database consists of 1200 face images of 450 children [15]. These datasets are never disclosed for research purposes.

Fourth, a face database of minors exist, which obtains and discloses face data with the consent of the guardians of the minors involved. The Children Multimodal Biometric Database (CMDB) is a database consisting of the iris, fingerprint, and face images of 106 children [16]. This database contains 1060 face images and is available for research purposes only.

Finally, there are face databases that obtain and disclose face data with tacit consent. The Cross-Age Celebrity (CACD) dataset and In The Wild Child Celebrity (ITWCC) dataset have been constructed by randomly collecting data from the internet. The CACD dataset consists of 163,446 face data of 2000 celebrities collected from the internet [17]. The ITWCC dataset consists of 7990 face data of 304 celebrities, collected from the internet [18]. These datasets may also delete data at the request of the target.

Among face datasets, the only publicly available face image datasets that include children between the ages of 2 and 18 years are, to the best of our knowledge, FG-NET [10] and FaceTracer [19]. The Cross-Age Celebrity Dataset (CACD) was built to evaluate face recognition performance with respect to aging [17]. However, this dataset did not include children under the age of 10, and only 199 participants were under the age of 18. The Children’s Longitudinal Face (CLF) dataset is not publicly available. This dataset collected at least four images from 919 children over an average of 4.2 years (total: 3682) [13].

The dataset collected in this study is a face dataset of children from whom face data has been obtained with consent of their guardians, but the data is neither disclosed nor available for distribution. In order to recognize a child’s face in real time, face data in various directions are required. In this study, data were collected with a total of four cameras. Simultaneous photography from multiple directions is possible with limited resources by using four cameras at once. Because it is difficult to control the movements of children, and it is not right to forcibly restrict them, it is also not good to photograph children for a long time to document various types of facial data. It is possible to extract varied information such as color distribution, dominant color, and the overall texture even from a static image. However, when collecting static images with our resources, we have no choice but to collect only four images at a time. All of our datasets were collected by video. When taking a video, various images can be obtained in a short time. By not limiting children’s behavior, more natural and diverse data can be obtained. Our dataset consists of data from 74 preschoolers between the ages of 2 and 7 years. We collected videos of less than 1 min per child, and a total of about 36 min of video data was collected.

## 3. Database

To collect children’s face data, children, parents, and guardians must be informed about exactly how this data will be processed and for what purpose, and consent must be obtained for this. In this study, after asking the childcare institution for cooperation in advance, an official letter was sent to parents and guardians informing them of the necessity of collecting the data and its purpose, and asking for their consent. In studies related to age, it is found that the longer the time between the collection of the subject’s face training data and the collection of the test image, the lower the face recognition rate [20,21,22,23]. In addition, the higher the age, the higher the face recognition accuracy rate. Therefore, in this study, both face data collection for the database and image data collection for testing were completed within two months.

### 3.1. Data Collection Considering the Age of the Subject

In the case of databases for children, different situations have to be considered, unlike for adults.

First, to obtain the children’s data, it is necessary to consider language proficiency with respect to the children’s age. There is a significant correlation between children’s language ability, attention, and concentration [24,25,26]. Children’s cognitive ability, attention, concentration, and focus are affected by language proficiency. The lower the child’s age, the lesser is their cognitive ability, attention, and concentration. Accordingly, the younger the child is, the more difficult it becomes to collect data through explanation or motion demonstration. Considering this, when collecting face data from children, it should be possible to obtain a certain amount of data from various angles at a time, in a short time span, even if the children do not move. In addition to simple explanations, movements and facial angles should first be demonstrated to attract interest and lead the children to perform the necessary postures and face angles on their own.

Second, children’s data collection requires a lot of preparation. The behavioral characteristic of feeling intimidated and nervous when encountering new and unfamiliar situations or people is called behavioral inhibition. According to Kagan’s study, behavioral inhibition is said to be related to a high level of negative emotions in infancy, that is, shyness [27]. It is said that the greater the shyness, the stronger the behavioral inhibition, and this behavioral inhibition is alleviated through various experiences. However, in the case of shy children, these various experiences are useless. Therefore, it is necessary to prepare time for children to adapt so that they can perform data collection smoothly by performing preliminary education, such as explaining why and how to do this through the cooperation of teachers who are familiar with children. In addition, it is necessary to go through the process of acquaintance with the children through several visits before data collection. To minimize the shyness of the children, it is recommended to collect face data of children multiple times for brief intervals. When collecting data, the child’s wariness decreases over time, so a better quality of data can be collected as data collection progresses.

Third, to collect the face data of children, a separate space for data collection and a communication ability to increase concentration in the children is required. A child’s attention and concentration continues to improve with age, affecting academic skills and information acquisition [28]. Children’s concentration decreases when they are with their peers. When children lose concentration, they become distracted, making it difficult to collect data. Therefore, data must be collected in an independent space with no other children around. In addition, the concentration of children should be increased by using positive, easy words and high-pitched voices.

Fourth, when collecting data, the facial expression and growth rate of children should be considered. Among the nonverbal clues, facial expression is the most direct way to grasp emotions, and it is the most powerful and complex signal, excluding language, among the means of conveying emotional state [29]. Since child language development is not perfect, it is important to consider their ability to recognize emotions through facial expressions, which are a non-verbal clue. To collect children’s data, it is necessary to carefully examine the children’s facial expression and consider the children’s condition. In addition, in the case of children, their expressions are more diverse and freer than those of adults, and their faces can change rapidly depending on the growth rate, hairstyle, and wearing of glasses. Therefore, in order to collect accurate face data of children, it is necessary to continuously or repeatedly collect and organize them.

### 3.2. Our Data Collection Method & Database Structure

**Figure 1:**
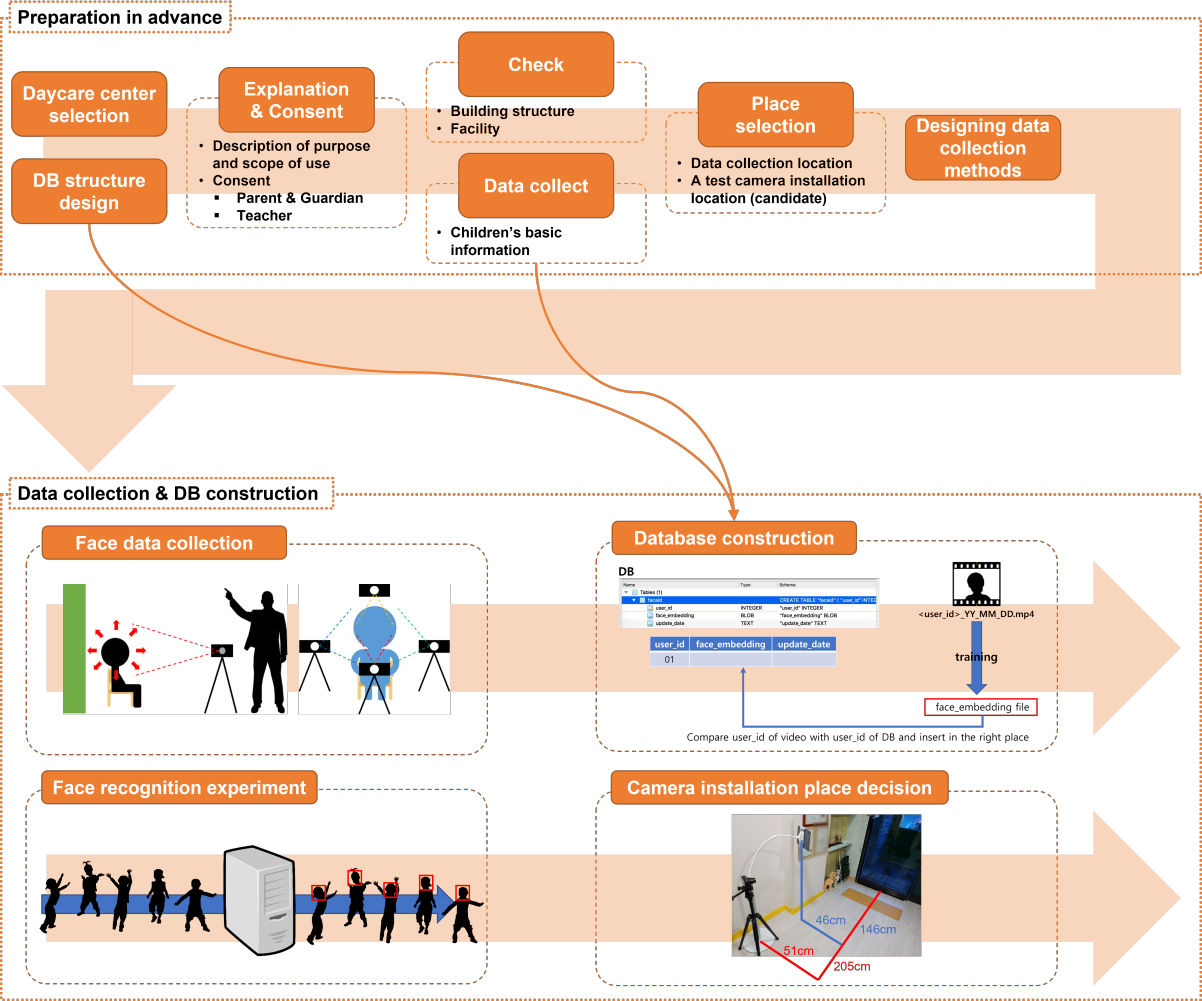
Data collection process flow chart.

In this paper, the face dataset of children was collected in two major steps, as shown in Figure 1. First, there is a pre-preparation process. In the pre-preparation process, after selecting a daycare center, consent is obtained from the teacher, parent, and guardian, and the building facilities are identified. First, the organization is selected by seeking cooperation from several organizations. Next, it is necessary to explain to teachers, parents, and guardians what data will be collected based on the DB (database) structure. It should also be clearly explained where and how these data will be used. Afterward, with the consent of the parents and guardians for data collection, the institution is visited to check the building structure and facilities. At this time, in order to facilitate data collection, each visit to the institution, with the help of teachers, builds intimacy with the children little by little. In the data collection and database construction stage, the child’s face data is collected at an independent space for data collection. In addition, the most appropriate location is found, after conducting the experiment using images taken from the location candidate group, to install the test camera. A database is built through the collected children’s face data and information.

Face recognition requires face data collected from various angles. For adults, one can get data from various angles by having them rotate their faces. However, it is difficult for children to move their faces on their own, like adults. As a result, in the actual test, even in the case of children between ages 5 and 7, some confusion occurred even if the exact direction was indicated. Therefore, in this study, when collecting the face data of children between ages 2 and 4, by changing the data collection method, data were collected through two methods according to the age of the child. Although two methods of collection were used, the results of the collected data were mostly the same. The data collection proceeded with the devices in Table 2.

**Figure 2:**
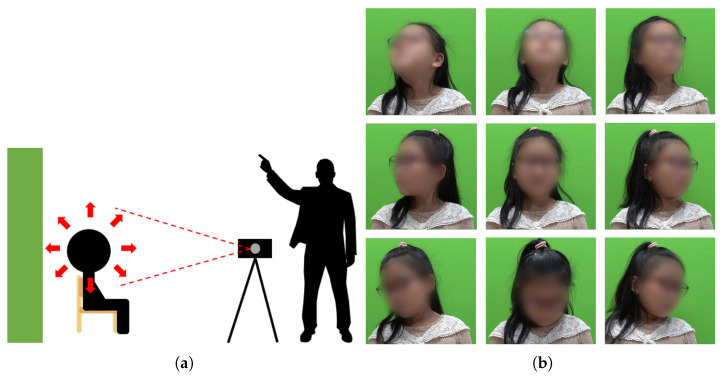
Face data collection for children aged 5 to 7: (**a**) camera position; (**b**) example.

In this study, 5-to-7-year-old children were instructed to move their faces in eight directions with words and actions, and videos were captured for one to two minutes (Figure 2). Children of this age seemed to be immature in their movements, but they generally tended to be cooperative and carried out adult instructions well. Most of the children of this age could move their faces in eight directions exactly according to instructions, so it was easy to collect data using only one camera. In this study, photography was conducted with a digital camera to collect good-resolution images of children’s faces. In addition, the distance between the child and the camera was set to about 1 m. The camera was installed as shown in Figure 2a, and face images were collected in eight directions as shown in Figure 2b.

**Figure 3:**
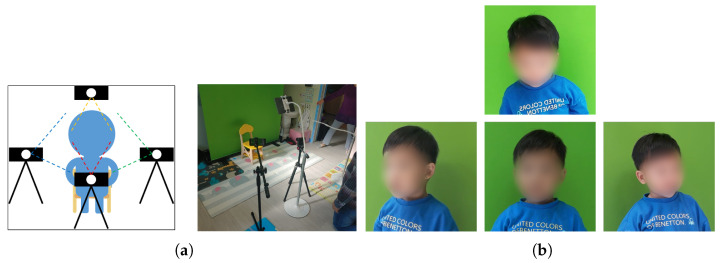
Face data collection for children aged 2 to 4: (**a**) camera position; (**b**) example.

Children under the age of 4 are still clumsy in words and actions. Therefore, it is difficult to collect face data in the same way as children aged 5 to 7. We decided to use four cameras for children under the age of 4 (Figure 3). When the child was in a designated position, images were taken from the top, bottom, left, and right using four cameras to obtain four directions of face data at a time. This method collected face data faster than the method of directing the motion of the child. In this study, two smartphones, one tablet PC, and one digital camera was used to collect the face data of children aged 2–4 years. To capture the children’s faces from various angles, smartphones were installed on the left and right sides, the tablet PC was installed in a structure overlooking from above, and the digital camera was installed in front of the child. Each camera was installed as shown in Figure 3a, and the distance between the child and the camera was within approximately 1 m. The children’s face data was collected in four directions as shown in Figure 3b.

In this study, face data was collected from a total of 74 children aged 2 to 7 years as shown in Table 3. All video data were shot with the highest resolution of 1920 × 1080. Video recording was performed from 30 s to 1 min per child. However, in the case of children aged 2 to 4 years, four videos were collected because the recording was performed with four cameras.

The structure of the completed database is shown in Table 4. In the database, the child is assigned a unique number (user ID). Next, the embedding data value of the learned face data and the time this value was calculated and loaded into the database are entered into the database. Since the embedding data value will be continuously updated with respect to the growth of the child, the update date is recorded in the database.

In this study, data correction, data deletion, and new data input were made possible through an auto-training program for continuous collection of children’s face data. In the auto-training program, when face data of a new child or new face data of an existing child are encountered, the results are uploaded to the DB after learning, and the DB is transmitted to the tablet PC. First, when new video data is entered into the auto-training program, user ID of the video was compared with user ID of the existing DB. At this time, the information of children, excluding the user ID and face learning data, is registered in the DB in advance by the person in charge, so it cannot exist if the user id of the video and the user ID in the DB is different. Finally, if embedding values exist when user IDs match, the calculated values are added to the back of the existing values and updated. If the user ID matches but no embedding values exist, the calculated embedding values are updated.

## 4. Appropriate Camera Positioning for Daycare Center

There are many things to consider when installing a camera in a childcare center.

First, problems such as camera loss must be considered, but concerns about invasion of privacy must also be prioritized. To collect children’s data, consent from parents and guardians must be obtained in advance. In addition, we must not infringe on portrait rights and privacy by photographing other people’s faces without permission. We need to select a location where the child’s face can be seen well, considering the above situation. This is related to the accuracy of children’s face recognition, so it needs to be tested several times.

Finally, according to the structure of the childcare facility, the camera should be installed at an appropriate location. The accuracy of face recognition and the usefulness of the system can vary depending on the location (indoor or outdoor) and height where the camera is to be installed. Children often express unpredictable behavior, such as walking backwards and saying hello, and so on. Therefore, camera installation for children’s face recognition should consider various environmental factors and child movement. In addition, since the structures of childcare facilities are all different, it should be considered that the location where children’s faces can be seen clearly may also change for each childcare facility.

### 4.1. Camera Installation Location for Daycare Center: Outdoor

For face recognition of children, a test image in which child registered in the DB appears is needed. To this end, we conducted an experiment to determine the camera position through a few tests. All experiments were conducted in a real environment of the daycare facility. In this study, we found a camera installation location for face recognition in an environment where children move naturally without stopping for a while in front of the camera. Rather than restricting movement, we thought it was appropriate to perform face recognition in a free-moving environment, given the characteristics of children, who are more likely to have atypical and unpredictable behaviors.

**Figure 4:**
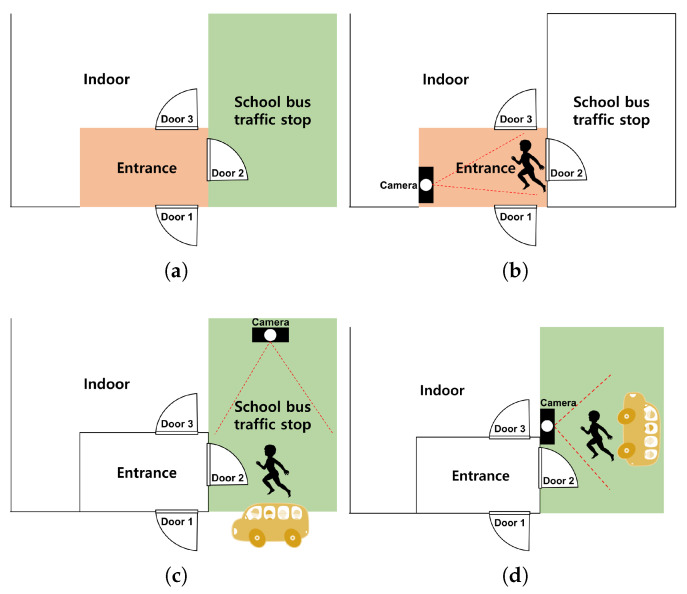
Camera installation location (outdoors): (**a**) outdoor structure of daycare center; (**b**) entrance; (**c**) school bus traffic stop; (**d**) door.

The outdoor structure of the childcare facility used for the test is shown in Figure 4a. We installed cameras at three locations and checked the video to see if the installation location was appropriate.

**Figure 5:**
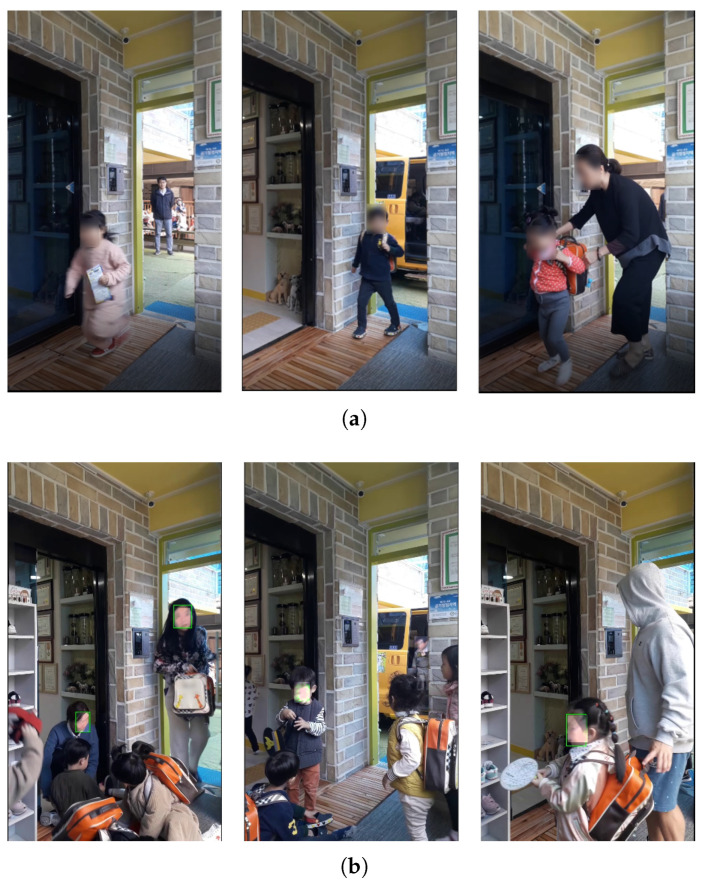
Example of an images taken with a camera installed at the entrance of the actual daycare center (Figure 4b): (**a**) best; (**b**) bad.

First, a camera was installed inside the entrance next to the school bus traffic stop, as shown in Figure 4b. This location is a door that must be passed through when a child gets off the school bus. For this study, the camera was installed at the height of the children’s faces in a place where the door was easily visible. If it had been installed in a structure that looked down from the top, considering the movement of children, there was a possibility that it would have been difficult to acquire the face data of the children; therefore, it was installed by selecting a location further inside. We conducted the experiment with a test video taken at this location. In this video, the detection was successful in 858 frames out of a total of 1012 frames, but the children’s face recognition rate was 29.03%. As shown in Figure 5b, in this location, most of the faces of teachers or guardians are photographed, or the child’s face is photographed at an angle of 45 degrees or more, so the face recognition was not good. The strengths and weaknesses of installing the camera in this location are listed in Table 5.

For this location, the distance between the children and the camera is small because the place is narrow. This is an advantage if we can detect the face of the children as in Figure 5a. However, if there are too many children in this location, it becomes difficult to shoot smoothly due to the crowded environment. In addition, in this location, the children’s movements were short, and only the backs of the children were often seen, so the face of the children was rarely seen in the recorded video (Figure 5b). Since this is an indoor location, which is less sensitive to external light, the illumination is constant, and the camera can be installed according to the height of the children. However, since the camera is installed within the reach of the children, there is a risk of frequent location changes and camera damage.

**Figure 6:**
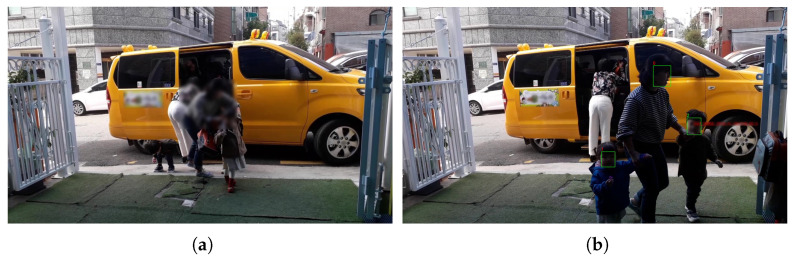
Images taken with a camera installed at the school bus traffic stop (Figure 4c): (**a**) example; (**b**) recognition result.

Second, a camera was installed outside the entrance next to the school bus traffic stop as shown in Figure 4c. We took a smartphone and filmed the children getting off the vehicle from the front. This is a location where children can check their faces with the camera right in front of them when they get off the school bus. In this study, the experiment was conducted with a test video of about 40 s taken at this location. In this video, the detection was successful in 850 frames out of a total of 1200 frames, but the children’s face recognition rate was 6.45%. In the filmed video, the children were too far, so face detection was not performed well in many cases, as seen in Figure 6a. In addition, the picture taken was too dark due to the awning on the ceiling, as seen in Figure 6b. The strengths and weaknesses of installing the camera in this location are listed in Table 5.

In this position, the children’s faces are captured well, and face detection performs well. However, the distance between the children and the camera is too far, and since it is outdoors, the illuminance may change significantly due to various environmental factors, so the children’s face recognition may not perform well. This location may seem convenient for face recognition because children’s movements are simple. However, the problem was that it was not easy to shoot because cars often stopped at places other than the designated school bus traffic stops. In addition, there were many adult interventions with children getting off and getting on the school bus.

**Figure 7:**
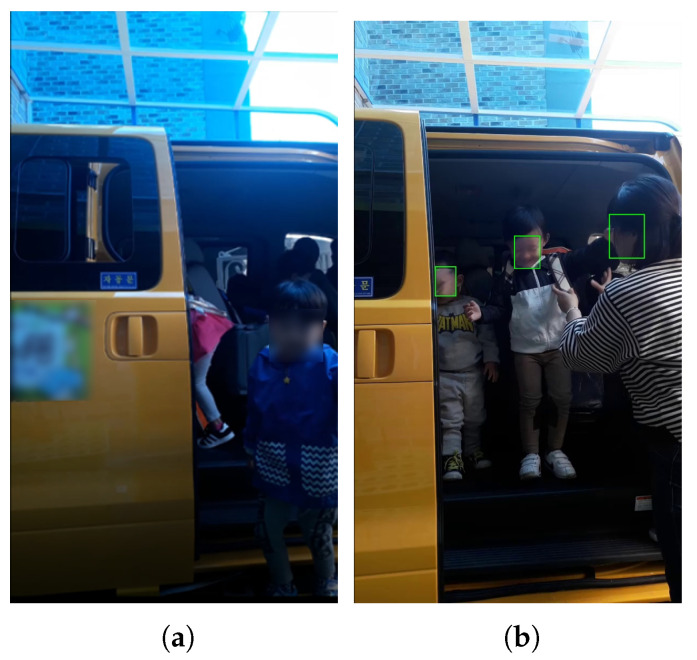
Images taken with a camera installed at door 2 (Figure 4d): (**a**) example; (**b**) recognition result.

Finally, a camera was installed at the entrance next to the school bus traffic stop, as shown in Figure 4d. This location is a door that children must pass through when they get off the school bus. The smartphone camera was installed to face the door of the vehicle. In this video, the detection was successful in 1109 frames out of a total of 4770 frames, but the children’s face recognition rate was 12.68%. Most of the videos shot were too dark, and the faces were filmed at an angle of 45 degrees or more, so it was difficult to recognize those who were filmed (Figure 7). The strengths and weaknesses of installing the camera in this location are listed in Table 5.

In this location, the space is not too crowded, and children move in an orderly manner compared to other locations. However, the distance between the camera and the children was too small, and the children’s faces were sometimes covered, depending on where they got off the bus. In addition, the school bus drop-off location changes frequently, and since it is outdoors, the illuminance changes are severe, so children’s face recognition may not be performed well.

Due to the characteristics of children, a lot of help from adults is needed, and the congestion is worse than expected when multiple entrances exist, as in the test location of this study. The camera for children’s face recognition should only shoot the child’s face, and it is right to exclude other people as much as possible. In addition, there should not be any factors that interfere with face recognition, such as a large change in illuminance or a face being covered. It should be considered that if a camera is installed outside, it can cause privacy infringement problems by photographing outsiders without prior consent. Therefore, to install a camera for face recognition outdoors, it is necessary to consider various external environmental factors.

### 4.2. Camera Installation Location for Daycare Center: Indoors

In this study, it is assumed that the optimal camera position to be used for the children’s face recognition test is a position facing the entrance from inside the room. If the camera is installed indoors, there is no need to fix the camera in one place because there is less risk of its loss. Although there is a risk of damage to the camera when moving the camera, problems such as distance, illumination, and space complexity can be controlled by moving the camera. The strengths and weaknesses of installing the camera indoors are listed in Table 5.

In this study, the camera was installed in five positions for four days to find the most suitable position for the children’s face recognition test.

**Figure 8:**
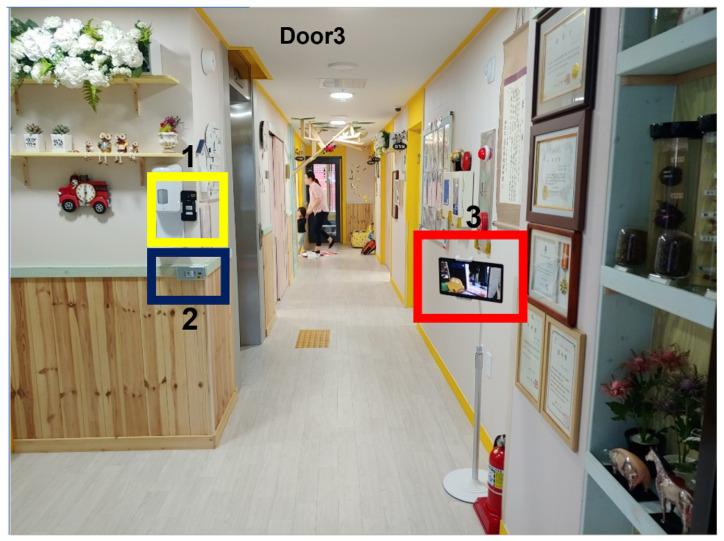
Installation of the camera indoors 1.

**Figure 9:**
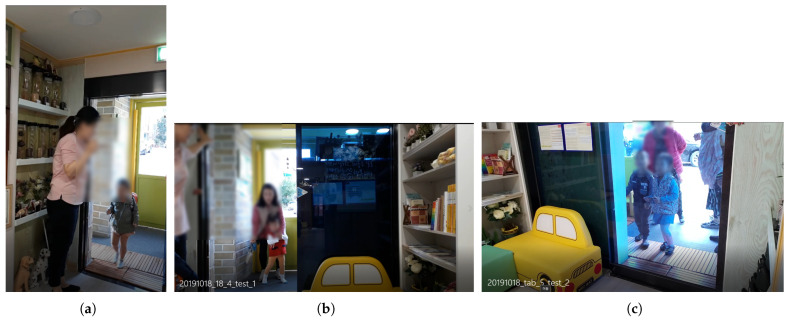
Actual captured images in Figure 8: (**a**) smartphone 1; (**b**) smartphone 2; (**c**) tablet PC.

First, as shown in Figure 8, one tablet (front camera) and two cellphones (rear camera) were installed. A tablet was installed on one side, and two smartphones were installed on the other side. By installing a smartphone at the top and bottom, respectively, we found the optimal height of the camera for face recognition. The test video was filmed for about two and a half hours, from 8:00 to 10:30 a.m., when children came to the daycare center. As a result of this setup installation, it was confirmed that among the three positions, the position where the tablet was installed had less intervention by adults and the face of a child was best seen (Figure 9). In this study, other cameras were installed in different locations based on the results in this location.

**Figure 10:**
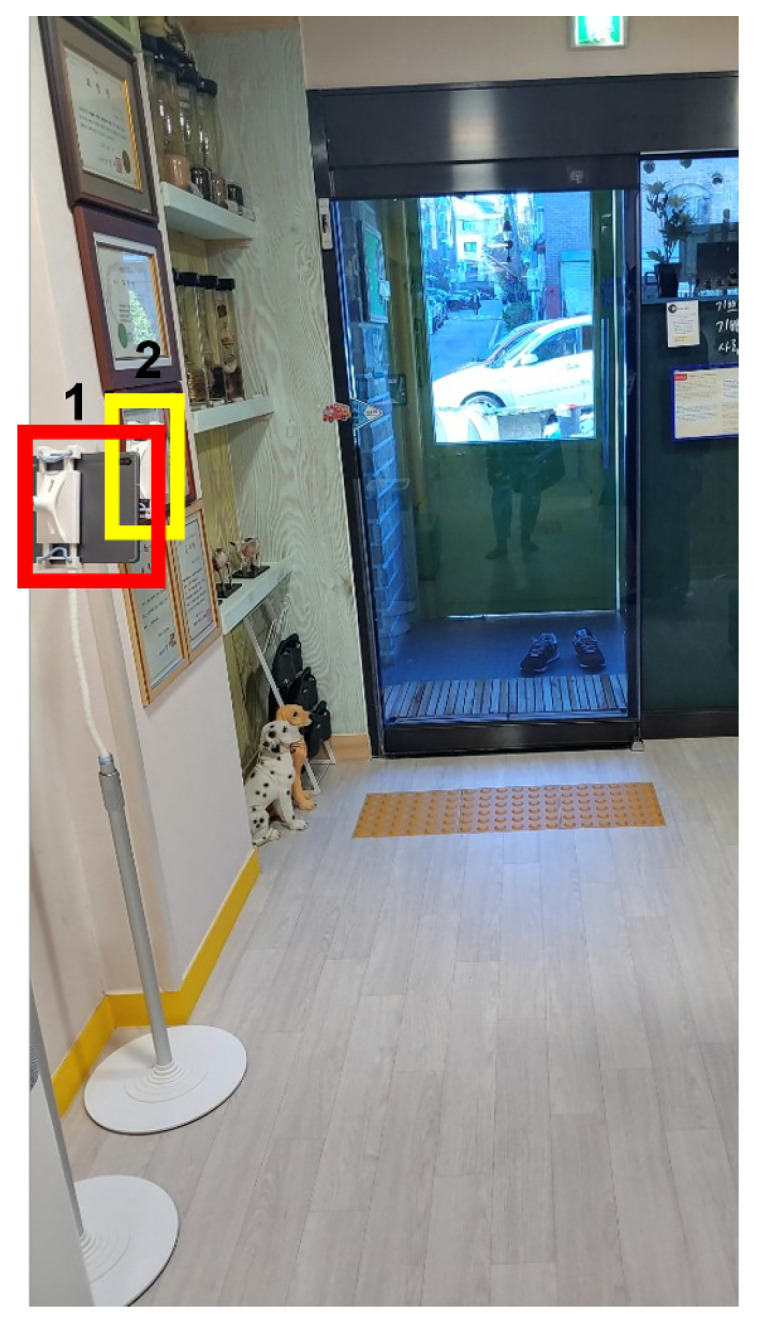
Installation of the camera indoors 2.

**Figure 11:**
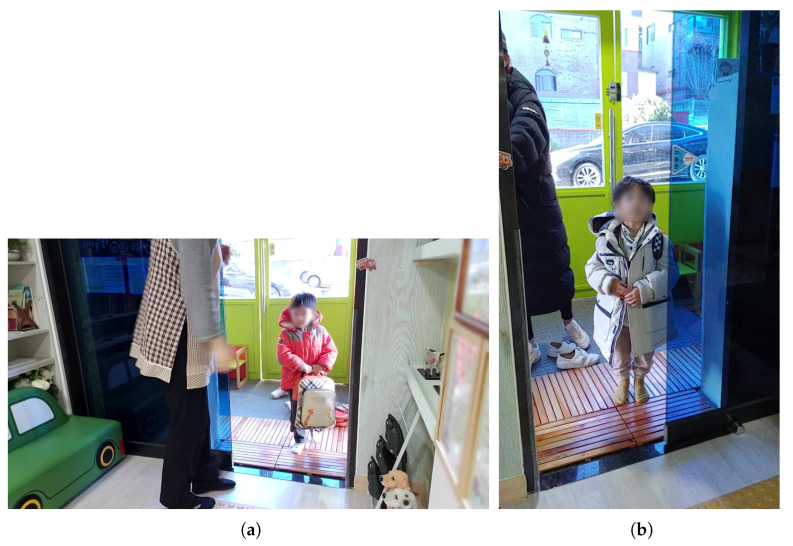
Actual captured images in Figure 10: (**a**) tablet PC; (**b**) smartphone.

Second, as shown in Figure 10, the camera of the tablet PC (front) and the camera of the mobile phone (rear) were installed in a position near the front of the entrance door. The test video was filmed for about two and a half hours, from 8:00 to 10:30 a.m., when children come to the daycare center. The camera installation results are shown in Figure 11. The front camera of a smart device is more effective in children’s face recognition than the rear camera because it can arouse children’s interest. When using a smart device’s camera, which uses a movable camera, the location may change or the camera function may be turned off when touched by children. However, if the front camera is used, children can see their reflection on the screen as they pass through the door, so the probability of securing the children’s front faces increases by arousing their interest.

**Figure 12:**
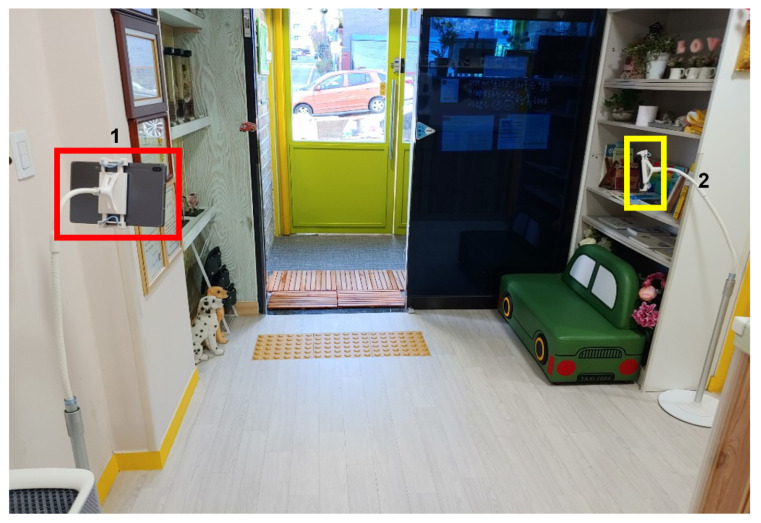
Installation of the camera indoors 3.

**Figure 13:**
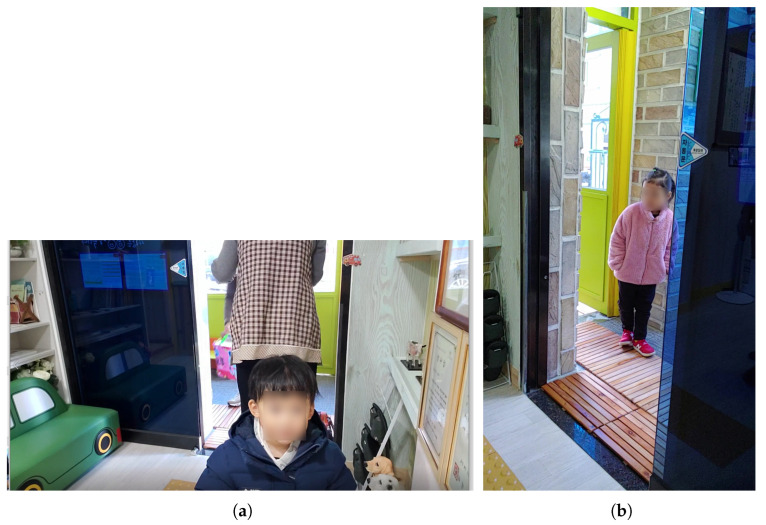
Actual captured images in Figure 12: (**a**) tablet PC; (**b**) smartphone.

Third, in this study, the tablet PC was installed on the left, and the smartphone was installed on the right, as shown in Figure 12. The test video was filmed for about two and a half hours, from 8:00 to 10:30 a.m., when children come to the daycare center. The camera installation results are shown in Figure 13. We assumed that by moving the camera to the right, it would be possible to shoot a part that could not already be seen based on where the existing tablet PC was installed. However, in this location, the children were often surrounded by teachers, and there were stairs next to it, which made it difficult for the children to move.

**Figure 14:**
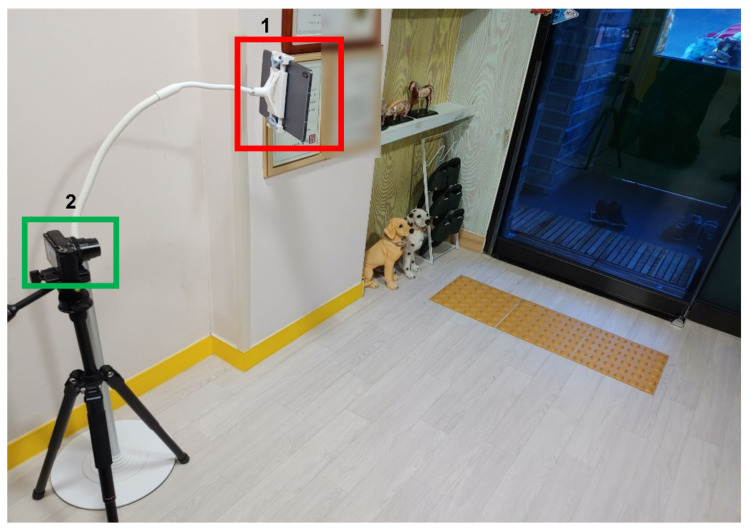
Installation of the camera indoors 4.

**Figure 15:**
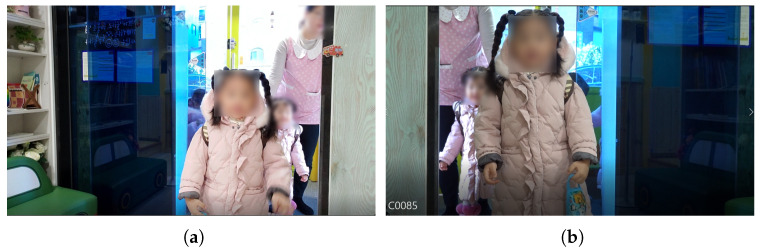
Actual captured images in Figure 14: (**a**) tablet PC; (**b**) smartphone.

Fourth, as shown in Figure 14, we installed the tablet camera and digital camera in similar locations. Through this test, we tested whether the face recognition rate varies depending on the resolution. In addition, we observed how we could better obtain the cooperation of children. It was observed which device of the two could better draw the children’s interest and obtain cooperation. The filming lasted about three hours, from 7:30 to 10:30 a.m. With this installation, the distance between the devices and the door was within 2 m, so the face recognition rate based on the resolution was meaningless (Figure 15). In the case of the tablet, since the child can directly see his or her face, it was able to attract their interest; hence, it was more suitable for face recognition than a general camera.

**Figure 16:**
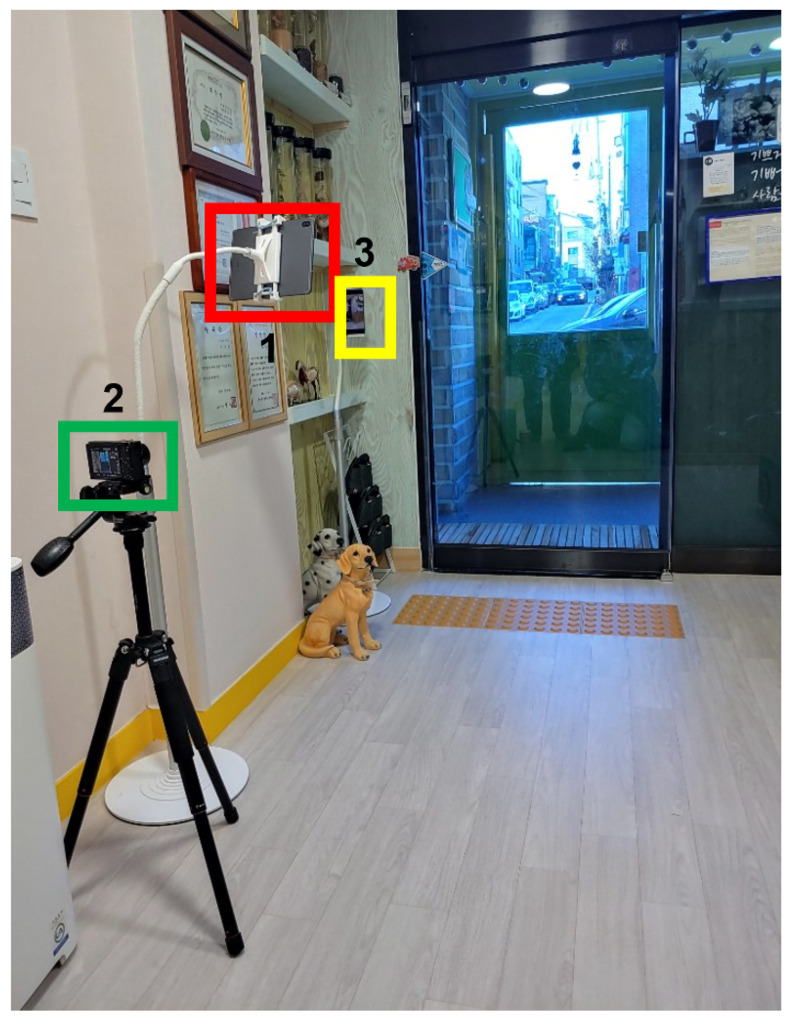
Installation of the camera indoors 5.

**Figure 17:**
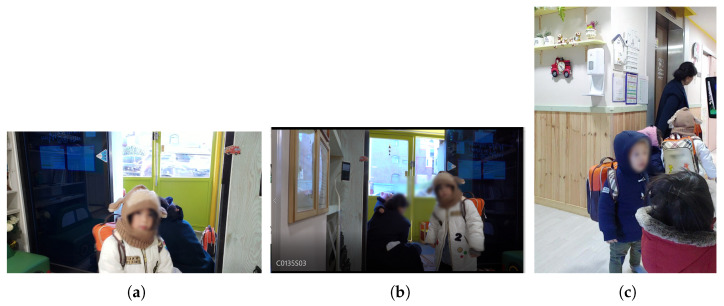
Actual captured images in Figure 16: (**a**) smartphone 1; (**b**) smartphone 2; (**c**) tablet PC.

Finally, the tablet and camera were installed in the same location as in the third test, and another smartphone rear camera was installed next to the door (Figure 16). The smartphone’s rear camera was installed in consideration of children walking backwards while greeting their parents or friends at the door. The filming lasted about two and a half hours, from 8:00 to 10:30 a.m. The camera installation results are shown in Figure 17. In this set-up, the smartphone installation location was not appropriate because the teacher often covers the children’s faces while greeting them at the entrance. Therefore, it was confirmed that this location was not efficient because the child’s face was seen less relative to the filming time.

## 5. Experiment

In this study, several tests were performed to conduct an experiment to check the performance of the collected DB. To calculate the face recognition rate, we collected test images and conducted experiments through a combination of several algorithms.

### 5.1. Pre-Experiment for DB Performance Test

In this study, a recognition rate change experiment was performed with respect to the distance between the test camera and the child. For this experiment, among the videos in which only one child is seen, videos in which the child is far away and videos in which the child is closer were used.

In this experiment, 16 out of 100 faces were correctly recognized in the far away videos, and 792 out of 1000 faces were correctly recognized in the nearer videos, as shown in Table 6. Unlike the far away videos, in the test images of nearer videos, the recognition rate may have increased because only one child is seen. However, considering that the face recognition failure rate decreased from 48% to 1%, the recognition rate increased as the distance between the camera and the child decreased. This means that the distance between the camera and the child affects the recognition rate. Through this experiment, we found that the distance between the camera and the child (within 1.5 m) is important and that this distance affects the recognition rate. To install a camera for face recognition in a space where children exist, the following should be considered. In a challenging environment, as in this study, it is necessary to find a location that strictly satisfies the following conditions:

- The camera should be installed at a location such that it does not impede the movement of children.
- There should be few external elements that cover the children’s faces.
- Cameras should be installed where children’s movements are simple.
- Cameras should be installed in spaces that are not too crowded.
- The distance between the children and the camera should not be too close or too far (within about 1.5 m).
- A place where there is little involvement of teachers or guardians is preferable.
- There should be little risk of camera repositioning or breakage.
- The illuminance should be constant.

In this study, based on the results of several camera installation tests, the camera was installed indoors at the eye level of children. In the case of the daycare facility where our tests were conducted, it was not suitable to install the camera outdoors because there were many entrances. We installed the camera indoors, where there is minimal adult intervention and the distance between the camera and the children is not too far apart. We also found through several experiments that children are interested in seeing themselves through the camera. We used the front camera of a tablet PC, not a smartphone or a general camera. Because children can see how their faces are being filmed, they focus on the camera. Next, we made an application for the tablet PC that satisfies the following conditions to prevent problems such as closing the camera when children touch the screen:

- There should not be any visible buttons on the screen when the camera is running.
- The physical buttons should not work when the camera is running.
- The camera can only be terminated through a ‘hidden button’ (e.g., touch a certain position of the screen five times, etc.).

### 5.2. Algorithm

We conducted experiments in the following environments to test whether the algorithm fits our data:

- Operating system: Windows 10;
- CPU: Intel(R) Core(TM) i7-8700K;
- RAM: 32GB;
- GPU: NVIDIA Titan RTX;
- Programming language: Python 3.6;
- Machine learning library: PyTorch 1.8.1.

In this study, the following three tasks are combined and implemented as a final face recognition algorithm:

- Face detection: Retinaface;
- Feature extraction: MobileFaceNet + ArcFace;
- Classification: k-nearest neighbor (kNN).

**Figure 18:**
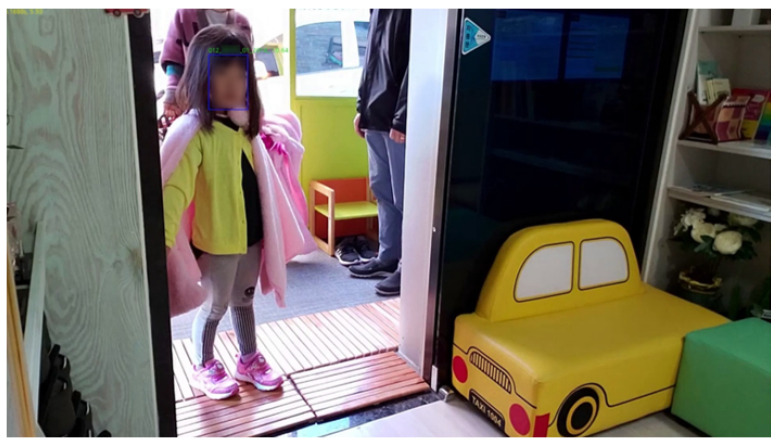
Test video used in experiment 2.

We used RetinaFace [30,31] as a face detector. They performed an experiment comparing face recognition rates when using Multi-task Cascaded Convolutional Neural Networks (MTCNN) [32] and RetinaFace as a face detection. As a result of the experiment, the recognition rate was higher when using RetinaFace than with MTCNN. In this study, a simple experiment was conducted to compare the recognition performance of MTCNN and RetinaFace with a short video of about 6 s in which only one child is present (Figure 18). In the experiment, only subjects with a visualization threshold of 0.5 or higher were considered faces. Table 7 shows that RetinaFace has a higher face detection success rate than MTCNN. Based on the results of this experiment, we used the RetinaFace algorithm to find the landmark of the face and align the face image.

In this study, MobileFaceNet was used as the feature extractor [33], and ArcFace was used as the loss for learning the feature extractor [30]. We used MobileFaceNet with a fast speed of calculation to recognize children’s faces in real time. MobileFaceNet shows an average of 17 FPS in the video with one child. We characterized these values by calculating the embedding values of each image of the face using the ArcFace algorithm. This ArcFace is used as a loss for learning MobileFaceNet, a feature extractor. Then, the calculated facial features were classified through a classification algorithm, k-nearest neighbor (kNN), and face recognition was performed.

### 5.3. Child Face Recognition Rate

In this study, the children’s face recognition rate was tested using the collected children’s face data and the test video.

**Figure 19:**
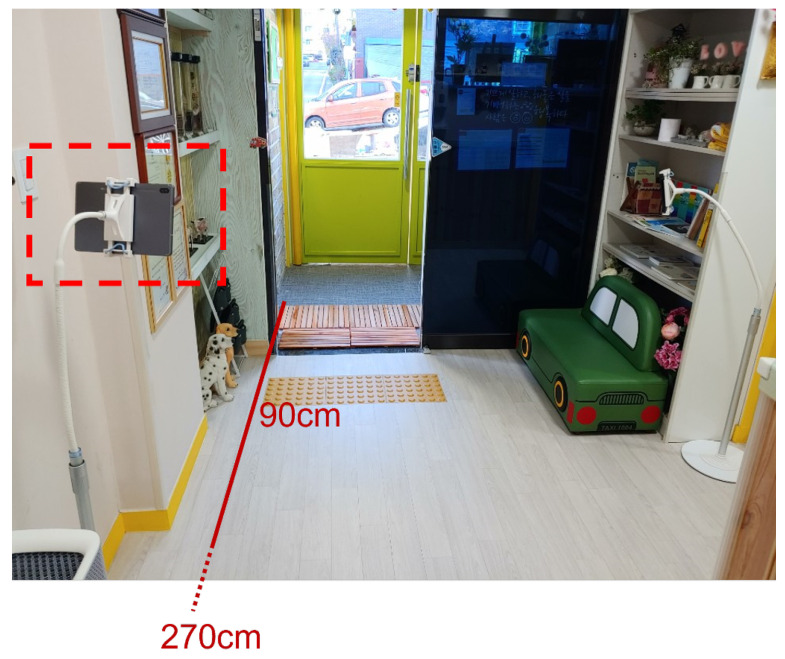
The location of the camera for taking the test video.

The videos for the experiment are videos recorded on a tablet PC for about 3 h and 10 min during the time when children come to the daycare facility. The tablet PC used to collect the video is about 0.9 m away from the door, and the maximum distance for face recognition of a child is 2.7 m, as shown in Figure 19. The collected video was edited and cropped down to 20 min and 45 s, and a total of 17 videos were created. Table 8 shows the number of children appearing in each video and the length of each video.

The face recognition rate depends on the distance value (embedding score) between the embedding vector value of the target and the embedding vector value of the training data. The embedding score is calculated through the Euclidean distance of the kNN algorithm. The closer this score is to 0, the more similar it is to the face data used for training. When the test video is taken in an environment similar to the training data (photographed distance, image quality, lighting, movement, etc.), the embedding score value approaches 0. However, since it is not possible to keep such an environment completely the same, we conducted an experiment to find the appropriate range of embedding score values for our DB (Table 9). In the experiment, face recognition was performed only when the size of the face image found in the video was 70×70 or more. With this setting, things that are too far away to be recognized are excluded. We performed an experiment to find the optimal embedding value threshold through the collected 17 videos, and calculated the face recognition rate in each image.

The range of embedding values is from 0 to 1.0, and the closer this value is to 0, the more it matches the DB we built. In the experiment, the children’s face recognition rate was highest when the embedding value was 0.7 (Table 9). The reason that the recognition rate of image index 17 is close to 100 is that there is no data when the child applies the mask. Therefore, all of the results were categorized as ’unknown’ because the actual test did not recognize the face well. In addition, the younger the children, the more unclear the facial features are, so performing face recognition results in a lower recognition rate.

## 6. Conclusions

Face datasets target adults and celebrities. These datasets include those that disclose and distribute databases with consent from those who provide face data, and those that obtain and disclose face data with tacit consent. Other databases are mostly private. In this paper, consent from parents or legal guardians was obtained for the collection of children’s data. However, to protect children’s personal information, it is used only for research purposes and is not disclosed to the outside world.

In this study, a database was established for collecting face data to be used for children’s face recognition. In addition, we presented the points and methods to focus on while building a database for children, and looked at effective camera installation location for face recognition of children. In order to collect children’s data, various factors such as children’s language ability, attention, concentration, and shyness, as well as the collection space, should be considered. In addition, it is recommended that all data be private. In this paper, data were collected by simultaneously photographing children from multiple directions with a total of four cameras. At this time, more natural and diverse data can be obtained by not placing restrictions on children’s behavior. As face data collected from various angles are required for face recognition, this study collected data in two ways according to the age of children by changing the method of collecting face data according to age. In this study, considering various factors, we built a database by collecting face data of 74 children aged 2–7 years in a daycare center. We collected videos of less than 1 min per child, and a total of about 36 min of video data were collected. In this study, after installing the camera both outdoors and indoors, the optimal location was found, and as a result of the experiment, using the total of 17 test videos taken at this location, a recognition rate of about 70.82% was obtained.

For children’s face recognition research, it is necessary to build a database of children’s faces, and data collection should be done continuously for children who continue to grow. We believe that this study will be quite interesting for researchers in the same field, face recognition in general, and researchers in data on children’s faces. In the future, we plan to build a more complete children’s face database through continuous data collection.

## Figures and Tables

**Table 2:** Devices used to collect dataset.

**Smart Phone: Rear Camera**	Samsung Galaxy Wide 3
	Samsung Galaxy J3 2017
**Tablet PC: Front Camera**	Samsung Galaxy Tab S6 10.5
**Digital Camera**	Sony Cyber-shot DSC-RX100 VII

**Table 3:** Number of child face learning data collected.

	Age	7	6	5	4	3	2	Total
Gender								
Male		4	4	14	7	7	2	38
Female		4	6	8	14	3	1	36
Total		8	10	22	21	10	3	74

**Table 4:** Database structure.

Database Variable	Explanation
user_id	Unique ID for an child
face_embedding	Child face embedding data
update_date	Child face embedding data upload date

**Table 5:** The strengths and weaknesses of each camera installation location.

Location	Strengths	Weaknesses
Entrance Figure 4b	The children’s faces are clearly visible. Illuminance is constant. The camera is installed at the child’s eye level.	The moving space is crowded. Children mostly appear from the back. The movement of children is too complex. The children and the camera are too close. Risk of camera damage due to a low position of installation.
School bus traffic stop Figure 4c	Many frontal images of the faces of the children are taken. The children’s movement pattern is simple.	The distance between the children and the camera is too large. Because it is outdoors, the illuminance changes owing to various environmental factors. Children do not necessarily come to this location. School buses do not necessarily stop at this location.
Door Figure 4d	The space is not too crowded. The children’s movement pattern is simple.	The distance between the children and the camera is very less. Because it is outdoors, the illuminance changes owing to various environmental factors. Children do not necessarily come to this location. School buses do not necessarily stop at this location.
Indoor	The camera can be moved around after installation. – It is possible to install a camera in a place where there is less change in illuminance. – The camera height can be set according to the height of the children. – It is possible to install a camera in a place where the movement of children is simple. – A camera can be installed where there are few external elements to cover the children’s faces. – The camera can be installed where there is little or no teacher or guardian intervention. There is little change in illuminance.	There is a high possibility that the camera will be damaged.

**Table 6:** Recognition rate experiment according to the distance between the camera and the child.

Video Type	True	False	Not Detect	Total Frame
Far away	16	36	48	100
Closer near	792	198	10	1000

**Table 7:** RetinaFace and MTCNN performance comparison.

Algorithm	Number of Faces Detected/ Total Number of Faces (Ratio)	FPS
MTCNN	139/194 (71.65%)	21.09
RetinaFace	171/194 (88.14%)	27.98

**Table 8:** Videos used in the child face recognition experiment.

**Video index**	1	2	3	4	5	6	7	8	9
**The number of children**	1	1	5	4	5	1	1	2	7
**Length (min:s)**	1:00	1:05	2:55	1:44	1:10	0:40	1:21	1:35	2:19
**Video index**	10	11	12	13	14	15	16	17	
**The number of children**	1	1	11	1	3	2	1	1	
**Length (min:s)**	1:28	0:36	3:54	0:39	1:06	0:41	0:50	0:54	

**Table 9:** Child face recognition rate (%) according to the range of embedding score values (bold text: The most recognition rate by video index according to the range of embedding score).

	Embedding Score	0.1	0.2	0.3	0.4	0.5	0.6	0.7	0.8	0.9	1
Video Index											
1		**70.18**	**70.18**	**70.18**	**70.18**	**70.18**	68.98	63.55	59.04	53.92	37.95
2		62.83	62.83	62.83	62.71	62.83	63.82	**64.80**	62.83	58.22	47.37
3		54.68	54.68	54.68	55.49	61.88	71.87	**77.88**	71.44	66.22	60.66
4		45.98	45.98	45.98	48.14	61.58	69.12	**72.61**	71.62	68.82	61.19
5		**76.97**	**76.97**	**76.97**	76.88	76.62	76.18	74.17	66.02	52.63	35.38
6		55.40	55.40	55.40	55.40	58.63	75.90	**83.81**	82.01	73.02	61.51
7		51.79	51.79	51.79	51.79	52.37	63.85	75.75	**82.5**	78.48	71.16
8		58.10	58.10	58.25	58.61	60.17	63.11	**66.89**	66.68	55.72	44.65
9		69.83	69.83	69.83	69.68	70.48	74.66	79.94	**81.00**	75.27	62.92
10		88.42	88.42	88.42	88.42	88.97	**89.08**	76.41	69.24	55.90	34.73
11		67.37	67.37	67.37	67.37	70.06	80.54	85.03	**91.62**	91.02	78.44
12		57.87	57.87	57.98	62.12	74.67	83.57	**85.67**	79.6	71.54	61.76
13		85.30	85.30	85.30	85.30	86.20	88.91	89.18	**89.81**	83.59	63.30
14		78.94	78.94	78.94	78.94	79.75	82.87	**84.05**	78.13	67.85	51.90
15		81.72	81.72	81.72	81.72	82.14	**83.16**	79.26	73.10	66.74	59.75
16		74.25	74.25	74.25	74.25	76.49	80.97	**86.57**	82.84	74.63	68.66
17		100	100	100	100	100	100	100	100	100	100
Average		62.09%	62.09%	62.1%	62.47	64.9%	69.29%	**70.82%**	68.81	62.82%	52.70%

## Data Availability

Not applicable.

## References

[1] Gligorijević J. (2019). Children’s privacy: The role of parental control and consent. Hum. Rights Law Rev..

[2] Srinivas N., Ricanek K., Michalski D., Bolme D.S., King M. Face recognition algorithm bias: Performance differences on images of children and adults. Proceedings of the IEEE Conference on Computer Vision and Pattern Recognition Workshops (CVPRW).

[3] Elharrouss O., Almaadeed N., Al-Maadeed S. LFR face dataset: Left-Front-Right dataset for pose-invariant face recognition in the wild. Proceedings of the 2020 IEEE International Conference on Informatics, IoT, and Enabling Technologies (ICIoT).

[4] Wang Z., Wang G., Huang B., Xiong Z., Hong Q., Wu H., Yi P., Jiang K., Wang N., Pei Y. (2020). Masked face recognition dataset and application. arXiv.

[5] Huang B., Wang Z., Wang G., Jiang K., He Z., Zou H., Zou Q. Masked face recognition datasets and validation. Proceedings of the Proceedings of the IEEE/CVF International Conference on Computer Vision.

[6] Huang G.B., Mattar M., Berg T., Learned-Miller E. Labeled faces in the wild: A database for Studying face recognition in unconstrained environments. Proceedings of the Workshop on Faces in ‘Real-Life’ Images: Detection, Alignment, and Recognition.

[7] Sengupta S., Chen J.C., Castillo C., Patel V.M., Chellappa R., Jacobs D.W. Frontal to profile face verification in the wild. Proceedings of the IEEE Winter Conf. Applications of Computer Vision (WACV).

[8] Yi D., Lei Z., Liao S., Li S.Z. (2014). Learning face representation from scratch. arXiv.

[9] Ricanek K., Tesafaye T. Morph: A longitudinal image database of normal adult age-progression. Proceedings of the 7th Int’l Conference on Automatic Face and Gesture Recognition (FG06).

[10] (2014). FG-NET (Face and Gesture Recognition Network) Ageing Database. https://yanweifu.github.io/FG_NET_data/.

[11] Deb D., Best-Rowden L., Jain A.K. Face recognition performance under aging. Proceedings of the IEEE Conference on Computer Vision and Pattern Recognition Workshops (CVPRW).

[12] Best-Rowden L., Jain A.K. (2017). Longitudinal study of automatic face recognition. IEEE Trans. Pattern Anal. Mach. Intell..

[13] Deb D., Nain N., Jain A.K. Longitudinal study of child face recognition. Proceedings of the International Conference on Biometrics.

[14] Best-Rowden L., Hoole Y., Jain A. Automatic face recognition of newborns, infants, and toddlers: A longitudinal evaluation. Proceedings of the 2016 International Conference of the Biometrics Special Interest Group (BIOSIG).

[15] Bharadwaj S., Bhatt H.S., Vatsa M., Singh R. (2016). Domain specific learning for newborn face recognition. IEEE Trans. Inf. Forensics Secur..

[16] Basak P., De S., Agarwal M., Malhotra A., Vatsa M., Singh R. Multimodal biometric recognition for toddlers and pre-school children. Proceedings of the IEEE International Joint Conference on Biometrics (IJCB).

[17] Chen B.C., Chen C.S., Hsu W.H. (2015). Face recognition and retrieval using cross-age reference coding with cross-age celebrity dataset. IEEE Trans. Multimed..

[18] Ricanek K., Bhardwaj S., Sodomsky M. A review of face recognition against longitudinal child faces. Proceedings of the International Conference of the Biometrics Special Interest Group (BIOSIG).

[19] Kumar N., Belhumeur P., Nayar S. Facetracer: A search engine for large collections of images with faces. Proceedings of the European Conference on Computer Vision (ECCV).

[20] Otto C., Han H., Jain A. How does aging affect facial components?. Proceedings of the European Conference on Computer Vision (ECCV).

[21] Klare B., Jain A.K. Face recognition across time lapse: On learning feature subspaces. Proceedings of the 2011 International Joint Conference on Biometrics (IJCB).

[22] Ramanathan N., Chellappa R. (2006). Face verification across age progression. IEEE Trans. Image Process..

[23] Grother P., Ngan M. (2013). Face Recognition Vendor Test (FRVT) Performance of Face Identification Algorithms.

[24] Bruce B., Thernlund G., Nettelbladt U. (2006). ADHD and language impairment. Eur. Child Adolesc. Psychiatry.

[25] Lonigan C.J., Bloomfield B.G., Anthony J.L., Bacon K.D., Phillips B.M., Samwel C.S. (1999). Relations Among Emergent Literacy Skills, Behavior Problems, and Social Competence in Preschool Children From Low- and Middle-Income Backgrounds. Top. Early Child. Spec. Educ..

[26] Willcutt E.G., Betjemann R.S., Wadsworth S.J., Samuelsson S., Corley R., DeFries J.C., Byrne B., Pennington B.F., Olson R.K. (2007). Preschool twin study of the relation between attention-deficit/hyperactivity disorder and prereading skills. Read. Writ..

[27] Kagan J. (1989). The concept of behavioral inhibition to the unfamiliar. Perspectives on Behavioral Inhibition.

[28] Blackman J.A., Westervelt V.D., Stevenson R., Welch A. (1991). Management of preschool children with attention deficit-hyperactivity disorder. Top. Early Child. Spec. Educ..

[29] Barrett L.F., Lindquist K.A., Gendron M. (2007). Language as context for the perception of emotion. Trends Cogn. Sci..

[30] Deng J., Guo J., Xue N., Zafeiriou S. Arcface: Additive angular margin loss for deep face recognition. Proceedings of the IEEE/CVF Conference on Computer Vision and Pattern Recognition (CVPR).

[31] Deng J., Guo J., Ververas E., Kotsia I., Zafeiriou S. RetinaFace: Single-Shot Multi-Level Face Localisation in the Wild. Proceedings of the IEEE/CVF Conference on Computer Vision and Pattern Recognition (CVPR).

[32] Zhang K., Zhang Z., Li Z., Qiao Y. (2016). Joint face detection and alignment using multitask cascaded convolutional networks. IEEE Signal Process. Lett..

[33] Chen S., Liu Y., Gao X., Han Z. Mobilefacenets: Efficient cnns for accurate real-time face verification on mobile devices. Proceedings of the Chinese Conference Biometric Recognition.

